# “Neptune Balls” Polysaccharides: Disentangling the Wiry Seagrass Detritus

**DOI:** 10.3390/polym13244285

**Published:** 2021-12-07

**Authors:** Lukas Pfeifer

**Affiliations:** Department of Pharmaceutical Biology, Pharmaceutical Institute, Faculty of Mathematics and Natural Sciences, Christian-Albrechts-University of Kiel, 24118 Kiel, Germany; lpfeifer@pharmazie.uni-kiel.de

**Keywords:** *Neptune balls*, *Posidonia oceanica*, seagrass, xylan, biofuel, reuse, polysaccharides, carbohydrate composition, ATR-FT-IR, GC-MS

## Abstract

Each year, high amounts of dead seagrass material are washed ashore at beaches world-wide. In the Mediterranean region, the seagrass *Posidonia oceanica* is responsible for huge agglomerates of ball-like seagrass litter. As these are often removed due to touristic reasons, a reuse method would be a step towards a more ecologically oriented society. In this study, the main polysaccharide components were analyzed, in order to propose possible usage options. To do this, different aqueous fractions were extracted, analyzed by classical carbohydrate analysis methods (GC-FID/MS, colorimetric assay and elemental analysis), and purified by ion-exchange chromatography, as well as selective precipitation with a detecting agent for highly glycosylated glycoproteins. The obtained purified fractions were analyzed in detail and a linkage-type analysis of the most promising extract was conducted via permethylation. Only low amounts of glycoproteins, as well as medium amounts of the characteristic apiogalacturonan were likely to be present, while xylan seemed to be the most abundant polysaccharide in most fractions. A partial structural proposal showed general accordance with land plant xylans, presenting reuse options in the field of biofuel and bioplastic generation.

## 1. Introduction

Visitors of Mediterranean coasts may have seen brown, fibrous balls—so-called Ægagropili or *Neptune balls*. Although they show structural similarity [1] to the green algae *Aegagropila linnaei* (syn. *Cladophora aegagropila*), they are, in contrast to that, dead plant parts of the angiosperm plant *Posidonia oceanica* (L.) Delile. This endemic seagrass species is a member of the sole genus in the family of *Posidoniaceae* and forms wide and dense meadows [2] across the Mediterranean Sea. While most members of this seagrass family are only found in Australia, the species *P. oceanica* occurs uniquely in the Mediterranean Sea [3]. Large seagrass beds are considered one of the world’s most valuable ecosystems [4], and in the case of *Posidonia oceanica*, the dead seagrass parts—also called necromass—are important for its ecological value by providing the life basis for micro-invertebrates [5]. Being once the dominant seagrass species in that area, it is now heavily endangered—by invading algae (especially *Caulerpa* species, see [6]), decreasing water and sediment quality [7] and human coast use projects [8].

**Figure 1:**
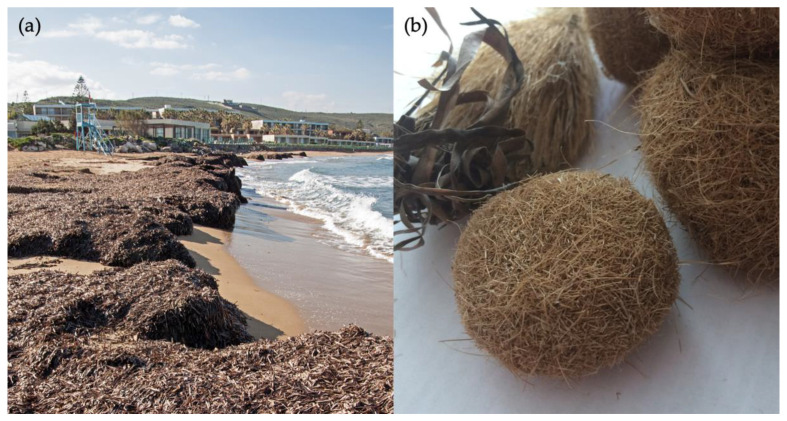
Dead seagrass material washed ashore. (**a**) A so-called “banquette” of *Posidonia oceanica* at one beach in Greece (photography owned by Dimitris Poursanidis; https://www.grida.no/resources/13408, last accessed 6 December 2021). (**b**) Investigated seagrass balls from Tunisia. Own photography by L. Pfeifer.

Human boats [6], mainly during the anchoring process (see [9,10]), stormy weather, and also the plant lifecycle itself lead to tons of leaf and rhizome material, which swims in the sea and frays out. Duarte [11] calculated the average amount of litter produced per year in 1 m^2^ of *Posidonia* meadow as 500 g dry weight. Due to wave motion, the most stable form is built [12], and therefore ball-like structures float to the beach (Figure 1).

The fibrous balls show some promising material properties, e.g., slow decomposition and low flammability. Because both features make them attractive as insulating material, this idea is being developed and pursued by some companies today [13,14]. Within this field of direct usage of the fibers, innovative ideas have arisen. These include, for example, their reuse as wall panels for offices, for smartphone cases and other lifestyle products, as well as compost for plant culture [15,16,17,18,19]. Furthermore, the idea of using the adsorption properties of the *P. oceanica* fibers for water cleaning falls into this category. Examples include, e.g., removal of dyes, phenols, and heavy metals [20,21,22,23,24,25,26,27].

In the last few years, another approach has become more relevant in the published literature on *Posidonia* balls: isolation and characterization of single molecular species [28] or groups of closely related molecules [29,30] in order to use them in subsequent steps (e.g., as biocomposites, polymer blends or bioactive extracts) or degrade them [31,32,33,34,35].

To the best of my knowledge, only one study [35] has looked specifically at the polysaccharide components in the *Posidonia* balls. Aqueous and solvent extracted fractions were compared and pharmacological activities were investigated. Polysaccharides are one of the most abundant groups of biopolymers in plant cell walls [36]. Knowing the composition of polysaccharides in the fibrous seagrass balls can enhance knowledge about long-term stability of the different groups of polysaccharides.

The aim of this study was to identify the structural characteristics of polysaccharide components in *P. oceanica* balls in order to suggest usage options for environmentally friendly products. Therefore, it is structured in two main parts: the first part characterizes some key components after fractionated cell wall extraction, while the second is focused on further purified extracts. Modern analytical methods (e.g., ATR-FT-IR, GC-MS) are applied to result in a partial structural proposal of xylan—the main polysaccharide component of the investigated fiber balls.

## 2. Materials and Methods

### 2.1. Plant Material

*Posidonia oceanica* “Neptune balls” were a kind gift of Prof. Dr. J. Woidasky (Pforzheim University). They were imported with permission from Tunisia by his collaborator Neptutherm GmbH (Karlsruhe, Germany). Before the extraction was done, the balls were roughly cut with scissors and milled in a laboratory grinder (IKA MF 10 basic with 1.0 mm sieve, IKA-Werke GmbH & Co.KG, Staufen, Germany).

### 2.2. Extraction Procedure

First an aqueous extraction of the ground plant material was performed, following the procedure of Pfeifer et al. [37]. Afterwards, a fractionated extraction modified from Raimundo et al. [38] and O’Rourke et al. [39] was used to isolate other major polysaccharide fractions. As described by Happ and Classen [40], the plant material was subsequently extracted with 0.2 M ammonium oxalate, 0.01 M hydrochloric acid, 3% (*w*/*V*) sodium carbonate and 2 M potassium hydroxide. Each extract was obtained after 24 h at 65–70 °C under constant stirring on a laboratory heater by vacuum filtration. Afterwards, it was neutralized, dialyzed (MWCO 12–14 kDa) and freeze-dried (Christ Alpha 1–4 LSC, Martin Christ GmbH, Osterode, Germany).

### 2.3. Geldiffusion Assay with Yariv-Reagent

To test the presence of arabinogalactan-protein glycan structures, a gel diffusion assay (compare [40,41,42,43]) was used. Therefore, β-D-glucosyl-Yariv [44,45] reagent was dissolved in a concentration of 1 mg/mL in double-distilled water. Then, 20 µL of that solution was pipetted in the middle hole of a perforated agar plate (1% agarose in 10 mM Tris-HCl buffer with 0.9% NaCl and 1 mM CaCl_2_, pH 7.3). Around this hole, three and four holes, respectively, were punched out and 20 µL of sample (high-molecular-weight fraction of *Posidonia oceania* seagrass balls in concentrations of 500 mg/mL, 250 mg/mL and 100 mg/mL) and *Zostera marina* HMF (100 mg/mL), as well as *Echinacea purpurea* AGP (10 mg/mL) as controls were pipetted in. After two days, the plates were checked for precipitation bands.

### 2.4. Purification with Yariv-Reagent

β-d-glucosyl-Yariv reagent was used for selective precipitation. Therefore, the absolute content of arabinose and galactose was calculated from the determined neutral monosaccharide composition of the aqueous extract. For detailed workflow, see [37].

### 2.5. Purification by Ion-Exchange Chromatography (IEC)

IEC purification was performed after solution of the sample in degassed water, which was then filtered (syringe filter, 0.45 µm, cellulose acetate, LLG GmbH, Meckenheim, Germany) and injected through 5.0 mL sample loop (Valve V-7, Pharmacia Biotech AG, Uppsala, Sweden) onto the column (Sepharose Q Fast Flow High Load XK 16/10; Pharmacia Biotech AG, Uppsala, Sweden). Before separation could be performed, the column was activated with 100 mL of 2 M NaCl solution and afterwards equilibrated with 100 mL of double-distilled water. With a step gradient (0.5 M, 1.0 M, 1.5 M and 2.0 M sodium chloride, each 50 mL), the applied sample was fractionated in portions of 10 mL volume. These were collected with the fraction collector Frac-200 (Pharmacia Biotech AG, Uppsala, Sweden) in sample tubes. Filtration and degassing of the solvents (Filtropur BT50, 0.1 µm, Sarstedt, Nürnbrecht, Germany) was carried out prior to use. A constant flow rate of 1.0 mL/min in the pump was set and the step gradient was performed manually. After fractionation, the colorimetric total monosaccharide determination method of DuBois et al. [46] was applied and the absorption was measured at 490 nm.

### 2.6. Neutral Monosaccharide Composition

Monosaccharide composition was determined by hydrolysis, reduction and acetylation following the general procedure of Blakeney et al. [47] with a modified hydrolysis step with 2 M trifluoroacetic acid. As internal standard, *myo*-inositol was used. The peracetylated pentoses, hexoses and desoxyhexoses were separated chromatographically by GC coupled with flame-ionization detection (Agilent 7890B, Agilent Technologies Inc., Santa Clara, CA, USA; column: Optima-225, 25 m, 0.25 mm, 0.25 µm; helium flow rate: 1 mL/min; temperature 230 °C; split ratio 30:1). A standard mixture of acetylated monosaccharides was used, peaks were identified via relative retention times and unknown peaks were identified by coupled mass spectrometer (MS: Agilent 5977B MSD, Agilent Technologies, USA).

### 2.7. Uronic Acid Determination

The content of uronic acids was determined colorimetrically according to Blumenkrantz and Asboe-Hansen [48]. For calibration a standard curve with different concentrations (5, 10, 25, 50, 100 µg/mL in 4% H_2_SO_4_) of a mixture (approx. 1:1, *w*/*w*) of glucuronic (GlcA) and galacturonic acid (GalA) was used.

### 2.8. Linkage-Type Analysis

Carbohydrate linkage types were determined following the procedure of Harris et al. [49]. Afterwards, the resulting partially methylated and acetylated monosaccharides (PMAAs) were determined using the exact GC-FID/MS instrument mentioned above. As a column, an Optima OV-1701-0.25 µm (Machery & Nagel, Düren, Germany) was used with helium flow rate of 1 mL/min and a temperature gradient from 170 °C to 210 °C with rate 1 °C/min, then a rate shift to 30 °C/min and a following hold time of 10 min at 250 °C. Masshunter workstation software version B.08.00 (Agilent Technologies Inc., Santa Clara, CA, USA) was used for analysis of the gas chromatograms.

### 2.9. Elemental Analysis

Combustion analysis was performed in the Institute of Inorganic Chemistry of Kiel University by use of a HEKAtech CHNS Analyzer (HEKAtech GmbH, Wegberg, Germany). The released gases were calculated by using the internal standard sulfanilamide.

### 2.10. FT-IR Spectrometry

For infrared spectrometric investigations, an IRAffinity-1S system (Shimadzu Corporation, Kyoto, Japan) was used together with the MIRacle 10 single reflexion ATR accessory (Shimadzu Corp). Spectra were obtained in the range from 600 to 4000 cm^−1^ with the resolution of 2 cm^−1^ by use of the LabSolutions IR software Version 2.10 (Shimadzu Corp).

## 3. Results

### 3.1. Fractionated Cell Wall Extraction

**Figure 2:**
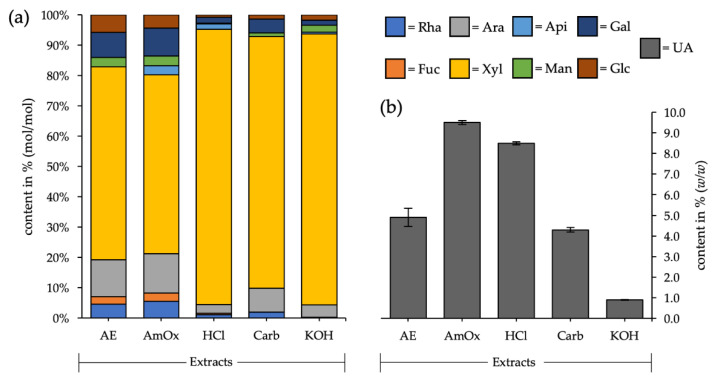
(**a**) Neutral monosaccharides and (**b**) uronic acid composition of the different aqueous fractions. AE: aqueous extract, AmOx: ammonium oxalate extract, HCl: hydrochloric acid extract, Carb: sodium carbonate extract, KOH: potassium hydroxide extract.

A fractionated cell wall extraction with different aqueous solvents was conducted in order to get an overview of the main groups of polysaccharides present in the remaining seagrass balls. The fraction with the highest yield was potassium hydroxide (KOH), accounting for approximately 57.5% (*w*/*w*) of all investigated fractions. Lowest in yield was the aqueous fraction (AE), at approximately 3.5% (*w*/*w*), while all other solvents resulted in extracts in the range of 5.9 to 17.8% (*w*/*w*). When looking at the monosaccharide composition of the different fractions (Figure 2; Table A1), there were some striking features:

Firstly, xylose was the major monosaccharide in all extracts, with a very high amount—between 59.0 and 90.8% (mol/mol). This is a strong hint for the presence of xylans (maybe arabinosyl- and/or glucuronosyl-decorated) as the main polysaccharide in the seagrass balls.

As a second observation, the presence of apiose and a higher amount of uronic acids in the ammonium oxalate (AmOx), as well as in the hydrochloric acid (HCl) fraction, was noticeable.

Thirdly, arabinose + galactose contents in the aqueous and in the ammonium oxalate extract were around 20% (mol/mol) and the ratio of arabinose to galactose was 0.7 in both cases. These are two key values to evaluate in the analysis of possible remaining arabinogalactan proteins in the seagrass balls.

The results of the elemental analysis (Table 1), performed on the different cell wall extracts, showed a content of 2.1 and 1.5% of nitrogen in the aqueous and ammonium oxalate extract, respectively. This corresponds (multiplied with 6.25) to an approximate protein content of 13.1 and 9.4% (*w*/*w*), respectively. The other three fractions showed a much lower elemental nitrogen content.

### 3.2. FT-IR Spectrometry

**Figure 3:**
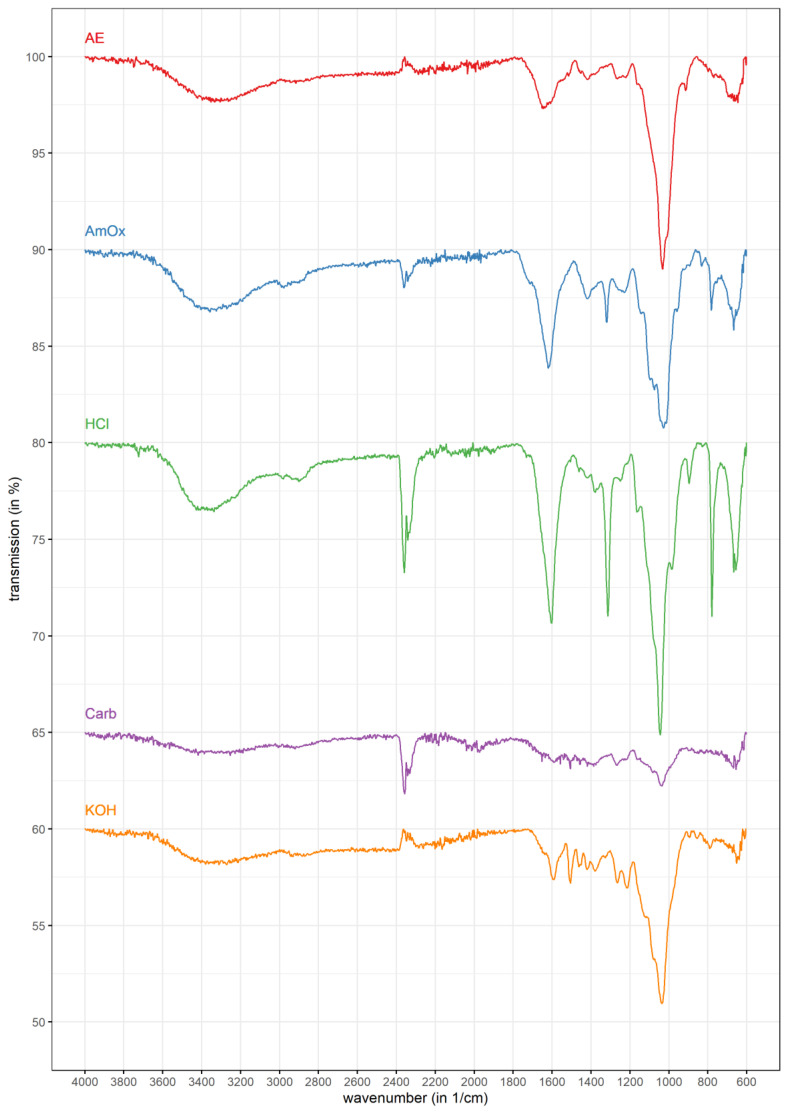
FT-IR spectra of the different polysaccharide fractions. For the upper three fractions, a transmission offset of 10% was chosen for visualization. For sodium carbonate and potassium hydroxide, offsets of 15% and 5%, respectively, were selected.

Attenuated total reflection (ATR) Fourier transformation infrared analysis (FT-IR) is a non-destructive method for sample characterization, commonly used in the field of quality control. To evaluate the findings of monosaccharide composition analysis in a broader context, it was performed for all extracts (Table 2, Figure 3).

The resulting spectra (Figure 3) showed overall similarity in most regions, but with different intensities (compare Table 2). In all spectra, one band was very intense, as from the very typical OH stretching signal from 3200–3400 cm^−1^.

It was in the range from 900–1100 cm^−1^ and was previously assigned by Robert et al. [55] to the β-1,4-linkage of xylan. In addition, another more intense band was visible between 1600 and 1700 cm^−1^. That, as well as the overall comparison of spectra, fits to the described characteristic (substituted) xylan regions according to Buslov et al. [50] (i.e., 1800–1500 cm^−1^, 1400–1200 cm^−1^, 820–780 cm^−1^ and 600–400 cm^−1^).

In most of the spectra, a peak around 2300 cm^−1^ was detectable. This could be assigned to CO2 from the measurement environment, an observation which is also supported by the small negative peak in that region for the potassium hydroxide extract.

### 3.3. Presence of Arabinogalactan Structures

**Figure 4:**
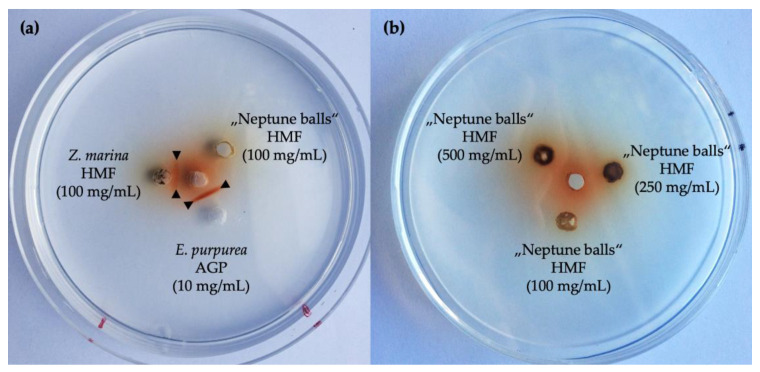
Gel diffusion assay of high-molecular-weight fractions of *Posidonia oceanica* seagrass balls. (**a**) As positive control, *Echinacea purpurea* AGP was used. For comparison, *Zostera marina* high-molecular-weight fraction was used. The black triangles highlight the bands formed by βGlcY and the samples. (**b**) Different concentrations of high-molecular-weight fractions of *P. oceanica* balls were used.

To investigate the presence of arabinogalactan proteins, a gel diffusion assay with β-Glc-Yariv (βGlcY) reagent was performed, which specifically interacts with AGPs by forming a clear precipitation band. The positive control *Echinacea purpurea* showed a strong interaction with βGlcY (Figure 4a), while the “Neptune balls” HMF on the same plate lacks this precipitation band. The same fraction of the seagrass *Zostera marina* showed a weaker, but clear interaction. Because this interaction depends on the present concentration, two higher concentrations were also used in this assay (Figure 4b). Neither of the showed clear interaction. Because the monosaccharides arabinose and galactose are present in the aqueous extract, it was possible to apply a classical AGP-isolation procedure by Yariv-precipitated extraction. Based on the total sugar yield, the necessary amount of βGlcY was calculated.

A precipitate was obtained, which was then further investigated and characterized for neutral monosaccharide composition (Table 3). The resulting precipitate was slightly enriched in the monosaccharides arabinose and galactose. Higher in content were the monosaccharides xylose and glucose, at 24.5 and 27.6% (mol/mol), respectively. By direct comparison of the high-molecular-weight fraction before precipitation and the non-precipitated part (called Yariv supernatant in Table 3), there was no clear difference, except in xylose and glucose contents.

### 3.4. Purification of Xylans from the HCl Fraction by Ion-Exchange Chromatography

The HCl fraction was chosen for further purification by ion-exchange chromatography (IEC), for two reasons.

**Figure 5:**
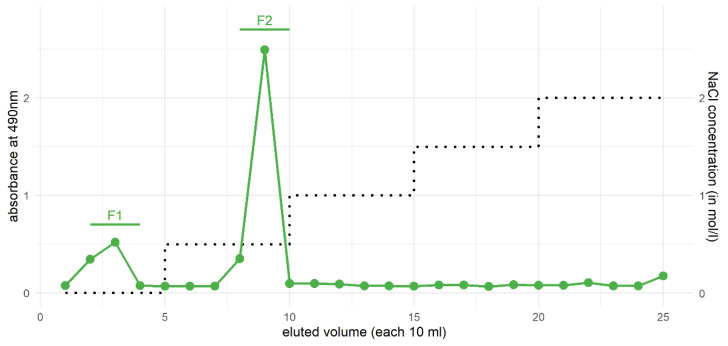
Ion-exchange chromatogram of the HCl fraction of *P. oceanica* balls.

Firstly, the amount of xylose was very high (Table 4), at more than 80% (mol/mol), and therefore a high amount of one molecular species was expected. Secondly, the fraction was visually white. This is a feature that makes purification through a gel column more equipment-friendly than from the potassium hydroxide fraction with its dark-brown color. In Figure 5—showing the obtained chromatogram of IEC analysis—two fractions are visually determinable. The first fraction between 10 mL and 40 mL eluted volume was very weakly bound, because it was eluted by pure water without sodium chloride addition. Between 80 mL and 100 mL eluted volume, the fraction with the highest absorption—corresponding with the highest sugar amount—was isolated. This fraction was only eluted by addition of 0.5 M sodium chloride.

By looking at the monosaccharide composition (Table 4), both fractions were very similar with their high amount of xylose. In the fraction F2, more monosaccharides other than xylose were present—uronic acids were higher, at 12.8% (*w*/*w*), than the crude HCl fraction.

### 3.5. Linkage-Type Analysis of Xylan Structures

**Figure 6:**
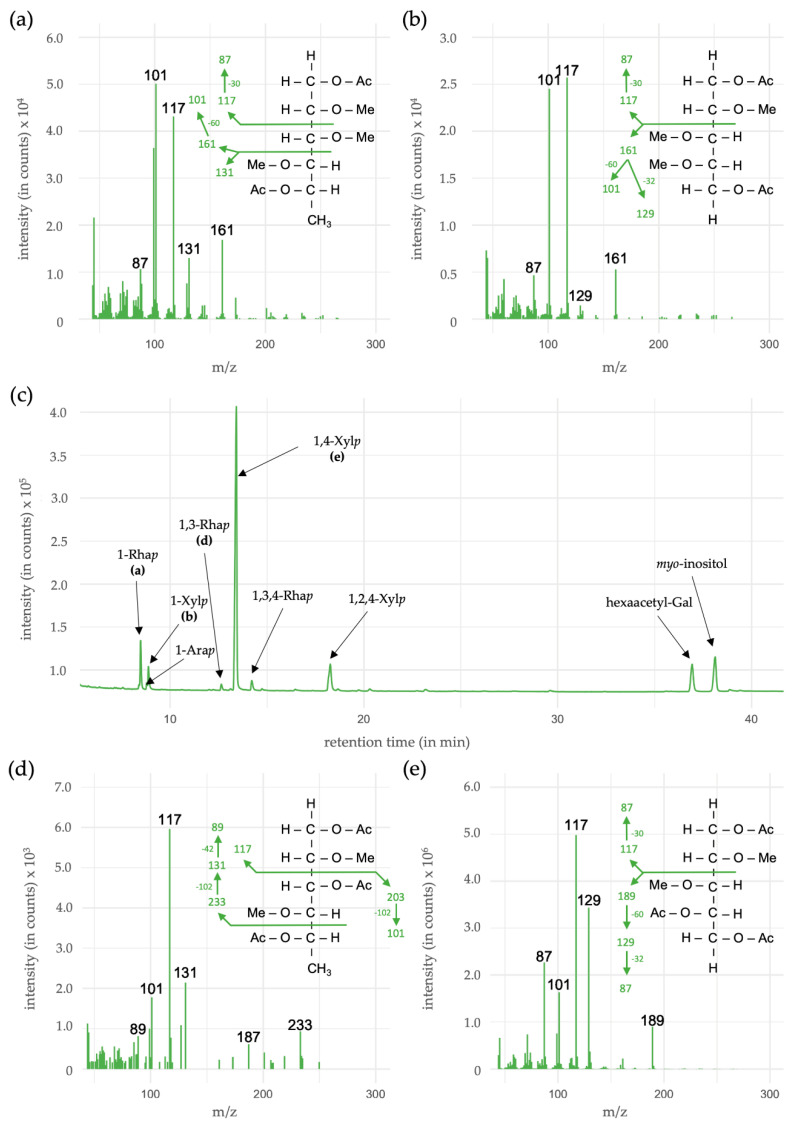
GC-results of the linkage-type analysis of the HCl fraction. In (**a**,**b**) and (**d**,**e**), the mass spectra of the highlighted peaks are given. In panel (**c**), the gas chromatogram over the relevant range of the investigated monosaccharides is shown.

In the linkage-type analysis, a methylation procedure of the HCl fraction was performed, followed by an acetylation step. With that workflow, the linkages in the samples were acetylated and all other hydroxyl groups of the carbohydrate were methylated. The resulting partially methylated alditol acetates (PMAAs) can be detected via their relative retention time, as well as their mass spectra by a combination of GC-FID and GC-MS (Table 5, Figure 6).

In total, the most occurring monosaccharide xylose made up 87.2% (mol/mol) of all linkage types in the HCl fraction, with 1,4-linked pyranosidic xylose as the dominating component. As 1,2-linked Xyl*p* and 1,4-linked Xyl*p* lead to similar PMAAs, it is not possible to distinguish between these two linkage types. As the occurrence of 1,2-Xyl*p* has not been described in such high amounts, it was not taken into consideration. The ratio of linear (1,4-Xyl*p*) to branched (1,2,4-Xyl*p*) was approximately 1:7.

Other linkage types were terminal-Ara, Rha and Xyl, as well as 1,3- and 1,3,4-linked Rha*p*. In the chromatogram (Figure 6c), one peak close to the internal standard is detectable. This was identified as undermethylated galactose and was therefore excluded from the linkage type table (Table 5).

## 4. Discussion

High amounts of seagrass rhizomes, roots, leaves and, in the case of *Posidonia oceanica*, also seagrass balls are washed ashore year by year. In most cases, this slowly decomposing material is removed due to beach care for touristic reasons.

Some works in the past few years have highlighted the value of the dead seagrass material—either in an ecological [5,56,57,58] or in a coast-protecting way [5,59]. Despite these values, this article deals with molecular characterization of the polysaccharide components in dead seagrass material, in order to suggest new options for the reuse of seagrass litter.

The amount of pectic polysaccharides was reduced, as estimated by the low uronic acid content in the ammonium oxalate and HCl fractions. These fractions usually provide most of the uronic acids of seagrass cell walls. A striking observation was the presence of apiose in both pectic fractions—a hint for the presence of apiogalacturonans (AGAs).

The cell wall extracts also suggested the presence of arabinogalactan(-proteins) (AGPs) from the results of monosaccharide composition analysis. These glycoproteins are important signaling components of the plant cell wall [60,61] and consist mainly of the pentose arabinose attached to a backbone formed of the hexose galactose. Recent investigations [37] have shown the presence of AGPs in *Zostera marina* and proposed the functionality of these molecules in salt adaption. Further attempts to identify AGPs with gel diffusion [62] assays and a full isolation procedure in this study showed no visible strong interaction in the seagrass balls. Pectins and AGPs are highly reduced in the seagrass balls thus underlining that these are less stable in comparison to, e.g., xylan.

### 4.1. Xylans

The most promising result of the fractionated extraction was the high xylose content (Figure 2, Table A1) in general, but especially in the HCl and potassium hydroxide fractions. That observation in combination with the low uronic acid content can hint towards the presence of (glucurono-)xylans—one group of the polysaccharide class of hemicelluloses. Extractability under alkaline conditions was formerly considered to be the main feature of hemicelluloses, but is questioned by some authors [63] with reference to the described solubility of, e.g., endosperm arabinoxylans in lower pH-ranges.

Xylans are hemicelluloses, which are after cellulose the second-most abundant group of plant cell wall polysaccharides [64]. While cellulose has a very conserved structure consisting of β-1,4-linked d-glucose units, xylan with its core β-1,4-linked d-xylose structure is more complex due to its variety of substitution patterns [65].

The highest xylose-containing fractions of the *P. oceanica* balls, namely the HCl and the KOH fractions, seemed to be ideal candidates for a further purification step by ion-exchange chromatography (IEC). Two fractions were achieved from fractionation of the HCl fraction, while one—the moderately charged second fraction from 80 to 100 mL eluted volume—made the quantitatively largest one. A moderate charge in the second fraction, as well as the monosaccharide composition also containing rhamnose, galactose and uronic acids in detectable amounts, supports the existence of *O*-acetylglucuronoxylans (AcGX) or arabinoglucuronoxylans (AGX). These xylan types described for eudicotyledons and gymnosperms [66] contain a so-called “sequence 1” at their reducing end [67]—a specific tetrasaccharide structure with 2 d-xylose-, one l-rhamnose- and one l-galacturonic acid units. Its biological function is still unknown [67], but it is proposed to have a functionality in length-regulation of the xylan chain [68]. Sequence 1 is present in many, but not all monocotyledon xylans [66].

FT-IR spectroscopy revealed one region broadly dominating around 900–1100 cm^−1^. The hypothesis of xylan being present in all fractions becomes more likely by comparing this exact region to literature. Ristolainen et al. [69] analyzed the influences of bleaching processes on xylan FT-IR spectra and also used a commercially available standard of xylan as reference. These spectra show obvious similarity to the obtained results of the Neptune ball spectra. The authors also concluded that extraction processes strongly influenced the number of additional bands, because of cleavage of possible substitution patterns [69]. Presence of signals from 3000 to 2800 cm^−1^ were also associated with presence of CH_3_-groups, being a hint for either methyl substitution or acetyl-groups [50]. Both are characteristic features of xylans [66]. In case of acety groups, the signal around 1740 cm^−1^ is also noticeable and should be taken into account (Table 2). These findings are in agreement with the monosaccharide composition, but also add more information about possible substitution. In *Zostera marina*, it has been shown [37] that the methyltransferases responsible for 4-*O*-modification of glucuronic acids, transferases from the DUF579 clade, are present. These were highlighted in the context of methyl decoration of AGPs, but were originally described as responsible enzymes for xylan modification [70]. Therefore, presence of methyl-substituted glucuronic acids in xylans of seagrasses seems to be likely.

Methylation analysis of the HCl fraction revealed a dominant presence of 1,4-linked xylose residues and smaller amounts of 1,2,4-linked xylose as branching points, being consistent with the proposed structures [67]. In addition, the ratio of linear to branched xylose was in the range of described xylans [71]. The latter branched xylose could also be a result of attached acetyl-groups, which are described for some dicotyledons, like *Arabidopsis* or *Populus* [66,67]. One disadvantage of our methodology was the lack of uronic acid detection. Even though they were not present in all described xylans, the determination in the crude fraction showed a presence of around 8.5% (Figure 2, Table A1). This could indicate occurrence as sidechains in possible glucuronoxylans. To further investigate these features, a carboxy-reduction prior to acetylation and methylation procedure should be performed [37,43].

As far as other sidechains are concerned, terminal arabinose is likely to act as decorating monosaccharide [67,71], sometimes present as smaller chains with attached xylose, ferulic or coumaric acid [72].

Coming back to xylan structures known from other plants, the so-called sequence 1 [66,67] consists of two xylose residues, in connection with one 1,3-linked rhamnose and one galacturonic acid. Uronic acids are difficult to cleave under mild acidic conditions (in this case 2M TFA) and remain connected to their linkage partners [73]. Therefore, the galacturonic acid is likely to stay linked to the terminal xylose (see above) and could not be detected in the analysis (see Figure 6). 1,3-Rha*p* is present in approximately 1.0% and could support the presence of this characteristic tetrasaccharide in connection to a xylan chain.

There are descriptions of sulfated polysaccharides from some green algae species, containing 1,3-Rha*p* residues and even 1,3,4-linked Rha*p* [74,75,76,77,78]. Therefore, an impurification with algae of the Neptune balls may be possible. To give more definitive structural statements, methodologies like xylan epitope profiling [79] or HILIC-MALDI-Tof/Tof-MS/MS [80] should be performed. Not only with regard to broadening the knowledge of plant biochemical composition, but also in the light of the reuse of the material, is a detailed structure necessary. For example, the most commonly used xylanases show a wide substrate specificity [71,81].

As far as other components in the seagrass balls are concerned, lignin has been described as being high (up to 44% of the dry weight) in that fibrous ball material [82]. Interestingly, lignin and hemicelluloses are often co-occurring, covalently bound as lignin-carbohydrate complexes, which are formed by oxidative coupling during biosynthesis [83,84].

### 4.2. Possible Usage Options

As the proposal of possible usage options for polysaccharides from *P. oceanica* balls is one of the goals of the structural analysis performed in this article, reuse should be the main focus with respect to xylan use.

Biofuel production is highly dominant in the field of the reuse of biological materials, referring to the production of fuels from non-fossil resources with regards to a more ecological balance [85]. This can be achieved either by degradation with special (thermophilic) bacteria [86] or chemical treatment [87,88]. Often, xylans are seen as disturbing factors in that process [89], but this issue has been solved by different xylan degrading enzymes [90,91]. For some seagrass species, the attempt to degrade them with bacteria has already been performed successfully [92]. In that study, *P. oceanica* showed the highest yield of lipids, but it was not stated whether seagrass balls were used. The xylose content of the investigated material was much lower and not in the focus of the degradation. One other study [93] investigated the bioethanol production from *P. oceanica* residues and showed high yield for that material. Therefore, in the direction of reuse as biofuels, some steps are already taken (also partly covered in the review by [94]).

Another option to evaluate is the acetylation reaction of the obtained xylans, making them thermoplastic [95,96]—an important feature on the way to “bioplastic” production. Recently, addition of hydrolyzed lignin to xylan biomass was evaluated to optimize the properties of xylan derived bioplastics [97]. With regard to high lignin contents [82] and the presence of xylans (this study), that idea sounds promising.

The chemical properties presented in this study could help to characterize a basic biomass, which is to be modified in order to obtain the needed properties.

## 5. Conclusions

The polysaccharide biomass in *P. oceanica* seagrass balls was investigated with different carbohydrate analyses to characterize them with regard to their proposed reuse as environmentally friendly materials. Most of the isolated polysaccharide moiety was composed of xylose-containing polysaccharides, mainly xylans. Other plant cell wall polysaccharides and cell wall proteins were degraded, and thus only present in very minor amounts. Usage of the so-called “Neptune balls” should therefore focus on xylan use. Direct isolation of (degraded) xylan from *P. oceanica* biomass is possible by extraction with dilute hydrochloric acid and additional purification steps by ion-exchange chromatography showed no strong differences in the monosaccharide composition. Therefore, the proposed workflow with dilute HCl is straightforward and cost-effective on the way to reuse the dead seagrass material, which naturally occurs at beaches worldwide.

## Figures and Tables

**Table 1:** Elemental analysis of the different extracts.

Polysaccharide Fraction	Nitrogen Content ^1^	Protein Content ^1^
AE	2.1	13.1
AmOx	1.5	9.4
HCl	0.1	0.6
Carb	0.8	5.0
KOH	0.2	1.3

^1^ All values are given in % (*w*/*w*).

**Table 2:** List of characteristic FT-IR bands obtained from the different cell wall fractions.

Wavenumber (in cm^−1^)	AE	AmOx	HCl	Carb.	KOH	Assignment	Literature Reference
3400	++	+++	+++	+	+	ν (O-H)	Buslov et al. [50]
3000–2800	+	++	++	+	+	ν (CH3), ν (CH2), ν (CH)	Bellamy [51]
2400–2300	+/−	+	+++	++	+/−	ν (CO2)	Bouilloud et al. [52]
1740	+	++	+	+/−	+/−	ν (C=O)	Szymanska-Chargot & Zdunek [53]
1600	++	++	+++	+	++	νasym (COO-)	Buslov et al. [50]
1500	+	-	-	+/−	++	δ (N-H) Amid II; ν (C=C-C) aromatic	Szymanska-Chargot & Zdunek [53]; Zhuang et al. [54]
1420	+	++	++	+	+	νsym (COO-)	Buslov et al. [50]
1300	+	++	+++	+	++	ω (CH2)	Zhuang et al. [54]
1250	+	+	+	+/−	+	ν (C-O)	Szymanska-Chargot & Zdunek [53]
1200–1000	+++	+++	+++	+	++	ν (C-OH)	Buslov et al. [50]
900	++	+	++	+/−	+/−	δ (C1-H)	Szymanska-Chargot & Zdunek [53], Robert et al. [55]

**Table 3:** Neutral monosaccharide composition of the different AGP-associated fractions and the aqueous extract (HMF). Standard deviation is given in brackets.

Monosaccharides	HMF ^1^	Yariv Precipitate ^1^	Yariv Supernatant ^1^
Rhamnose	5.5 (±1.1)	-	8.3 (±0.6)
Fucose	4.4 (±0.1)	-	5.7 (±0.2)
Ribose	-	-	1.6 (±0.2)
Arabinose	11.0 (±2.7)	18.3	11.4 (±0.2)
Xylose	26.4 (±1.8)	24.5	22.6 (±1.7)
Apiose	-	-	-
Mannose	8.2 (±0.6)	7.7	8.8 (±0.7)
Galactose	15.5 (±0.9)	21.8	16.5 (±0.6)
Glucose	29.0 (±3.2)	27.6	25.1 (±0.8)

^1^ All values are given in % (mol/mol). If standard deviation is given, the values were calculated as average from triplicates.

**Table 4:** Neutral monosaccharide composition of fractions obtained by IEC-fractionation of the HCl extracts. Uronic acids are given in % (*w*/*w*) and other monosaccharides are given in % (mol/mol).

Monosaccharides	F1	F2
Rhamnose	-	1.8
Fucose	-	0.9
Arabinose	-	5.3
Xylose	90.5	87.5
Apiose	-	-
Mannose	-	-
Galactose	-	3.6
Glucose	9.5	0.9
Uronic acids	n.d.	12.8

**Table 5:** Linkage-type analysis of the HCl fraction.

Monosaccharides	Linkage-Type	% (mol/mol)
Rhamnose (Rha*p*)	1-	8.4
	1,3-	1.0
	1,3,4-	2.4
Arabinose (Ara*p*)	1-	1.0
Xylose (Xyl*p*)	1-	4.8
	1,4-	72.0
	1,2,4-	10.4

## Data Availability

The data that support the findings of this study are available from the corresponding author upon reasonable request.

## Appendix A

**Table A1:** Neutral monosaccharide composition of the different aqueous fractions. All values are stated as average of triplicates in % (mol/mol).

Mono- Saccharides	AE	AmOx	HCl	Carb.	KOH
Rhamnose	4.5 (±0.7)	5.5 (±0.6)	1.1 (±0.2)	2.0 (±0.2)	0.2 (±0.2)
Fucose	2.5 (±0.4)	2.8 (±0.2)	0.5 (±0.1)	-	-
Arabinose	11.7 (±1.7)	12.6 (±2.7)	2.9 (±0.9)	7.8 (±4.4)	4.1 (±4.0)
Xylose	61.4 (±2.2)	56.9 (±1.6)	90.2 (±1.6)	81.8 (±4.6)	88.4 (±6.3)
Apiose	-	2.8 (±0.2)	1.8 (±0.3)	-	0.6 (±0.5)
Mannose	3.7 (± 0.7)	3.8 (±1.0)	0.2 (±0.3)	1.4 (±0.3)	2.7 (±0.7)
Galactose	9.5 (±1.5)	10.5 (±0.2)	2.3 (±0.3)	5.3 (±0.5)	2.0 (±0.8)
Glucose	6.7 (±0.7)	5.1 (±0.6)	1.0 (±0.2)	1.7 (±0.1)	2.0 (±1.5)
Uronic acids	4.9	9.5	8.5	4.3	0.9
